# Estimating cardiac output from coronary CT angiography: an individualized compartment model in comparison to the Stewart–Hamilton method

**DOI:** 10.3389/fcvm.2023.1156332

**Published:** 2023-11-20

**Authors:** Jon Bjarne Leiknes, Aksel Hiorth, Jorunn Havnen, Ole Jacob Greve, Kathinka Dæhli Kurz, Alf Inge Larsen

**Affiliations:** ^1^ Stavanger Medical Imaging Laboratory, Department of Radiology, Stavanger University Hospital, Stavanger, Norway; ^2^ Department of Clinical Science, University of Bergen, Bergen, Norway; ^3^ Department of Energy Resources, University of Stavanger, Stavanger, Norway; ^4^ Department of Electrical Engineering and Computer Science, University of Stavanger, Stavanger, Norway; ^5^ Department of Cardiology, Stavanger University Hospital, Stavanger, Norway

**Keywords:** computed tomography, angiography, contrast, cardiac output, model, estimate, Stewart–Hamilton, individualized

## Abstract

**Background:**

Attenuation is correlated with the concentration of contrast medium (CM) in the arteries. The cardiac output (CO) affects the concentration of CM in the circulatory system; therefore, CO affects the time–density curve (TDC). Thus, estimating CO using TDC from test-bolus images acquired in computed tomography (CT) is possible. In this study, we compare two methods of estimating CO, namely, an individualized mathematical compartment model, integrating patient, contrast, and scanning factors with TDC, and the Stewart–Hamilton method based on the area under the curve of the TDC.

**Materials and methods:**

Attenuation in the aorta was measured during test-bolus in 40 consecutive patients with a clinical indication for coronary CT angiography (CCTA). Each participant underwent cardiac magnetic resonance imaging following CCTA to validate the estimated CO. The individual compartment model used TDC in conjunction with scanning and patient-specific parameters to estimate the concentration of CM and CO over time. This was compared to the CO calculated from the area under the curve using the Stewart–Hamilton method.

**Results:**

Both CO estimated with our individualized compartment model (*r* = 0.66, *p* < 0.01) and the Stewart–Hamilton method (*r* = 0.53, *p* < 0.01) were moderately correlated with CO measured with cardiac MRI. Body surface area (BSA) and time to peak (TTP) affected the accuracy of our model. Lower BSA resulted in overestimation, and lower TTP resulted in CO underestimation, respectively. We found no gender-specific difference in the accuracy of our model when correcting for BSA. The Stewart–Hamilton method performed better with a more complete TDC, whereas the compartment model performed better overall with a partial TDC.

**Conclusion:**

The TDC acquired in CCTA allows for CO estimation. Both the Stewart–Hamilton method and our mathematical compartment model show moderate correlation when applied to our data, although each method has its strengths and limitations. If the majority of the TDC is known, the Stewart–Hamilton method may be more reliable, but an individual compartment model is preferable when there are insufficient data points in the TDC. Regardless, both methods can potentially increase the diagnostic information acquired from a CCTA, which is increasingly recommended in clinical guidelines.

## 1. Introduction

Coronary computed tomography angiography (CCTA) is increasingly recommended in clinical guidelines for chest pain assessment (1, 2), and strong evidence has established its role in the management and treatment of coronary artery disease (3, 4). The ability to reliably estimate cardiac output (CO) using simple parameters easily obtained directly from the CT images combined with the physical properties of patients would further increase the clinical value of CCTA, by providing an assessment of overall cardiac function in addition to evaluation of coronary artery disease.

Contrast enhancement in the aorta is measured in Hounsfield units (HU) at set intervals of time during the test-bolus series of a CCTA. Its primary purpose is to ascertain the timing delay of the main scan after contrast administration to ensure optimal image acquisition. The physical parameters of the computed tomography (CT) scanner are known, and the density of iodinated contrast is constant for a given voltage used for image acquisition (5). Thus, the change in contrast enhancement over time [the time–density curve (TDC)] is correlated directly with contrast agent concentration in the vessel, provided that the voltage remains constant.

CO plays a significant role in the distribution of contrast medium (CM) in the body and, subsequently, the achieved contrast enhancement in individual organs (5). It additionally provides information about the overall circulatory status of an individual and is useful information for the clinician referring a patient to CCTA.

Calculating the CO after image acquisition with prospective or retrospective electrocardiogram (ECG) gated techniques is possible but not with single heartbeat acquisition techniques as they lack both end-diastolic and end-systolic phases (6).

Single heartbeat acquisition techniques have been more prevalently used in clinical routine (7) and will likely continue to do so with further advances in scanner technology (8, 9).

The significant impact of CO on contrast distribution in the body (5, 10) may have implications for prospectively individualizing the CM protocol or adjusting the scan parameters for the CCTA examination.

Thus, the current study aimed to compare the CO estimation (CO_Model_) using an individualized compartment model (11) to simulate the distribution of CM in the body, with a Stewart–Hamilton method (12–14) utilizing the area under the curve of the TDC to estimate CO. The Stewart–Hamilton method is a purely data-driven approach, utilizing only the TDC, whereas the compartment model is personalized, utilizing patient-specific parameters such as height, weight, and gender.

The data available may vary during clinical application. For instance, part of the TDC may be unknown, if the test-bolus image acquisition concludes when the time to peak (TTP) of the concentration curve occurs and scan delay is ascertained. In other cases, patient-specific factors such as height or body weight may be unknown or could be extreme outliers.

By comparing the methods, we aimed to improve our understanding of the optimal application and limitation of each method to facilitate the clinical application of CO estimation from the test-bolus.

## 2. Materials and methods

### 2.1. Participants

In total, 40 patients who were referred to a CCTA with a clinical indication and scheduled accordingly at our department were included consecutively. Demographic information was obtained through empirical measurement, interviews, and from the medical records of the participant.

Patients with suspected myocardial ischemia were included. We excluded patients with previous myocardial infarction, heart failure, myocarditis, pericarditis, or valvulopathy.

### 2.2. Premedication prior to image acquisition

Premedication with beta-blockers and sublingual nitroglycerine was performed according to clinical routines. In participants who were not already prescribed beta-blockers with no contraindications, beta-blockers were administered orally 2 h prior to CCTA and intravenously at the CT laboratory if necessary, to ensure a target resting pulse of approximately 60 bpm. Cardiac magnetic resonance imaging (MRI) was performed immediately after CCTA to ensure that the dose of beta-blocker was sufficiently similar to CCTA. The participants received two puffs (0.4 mg/dose) of sublingual nitroglycerine immediately before the main CCTA examination and immediately before cardiac MRI, provided that they had no contraindications.

### 2.3. Image acquisition

#### 2.3.1. CCTA

CT images were acquired with a Siemens Somatom Definition Flash (Siemens, Erlangen, Germany, 2012). The chosen CM was Omnipaque 350 mg_Iodine_/ml (GE Healthcare, Oslo, Norway).

**Figure 1:**
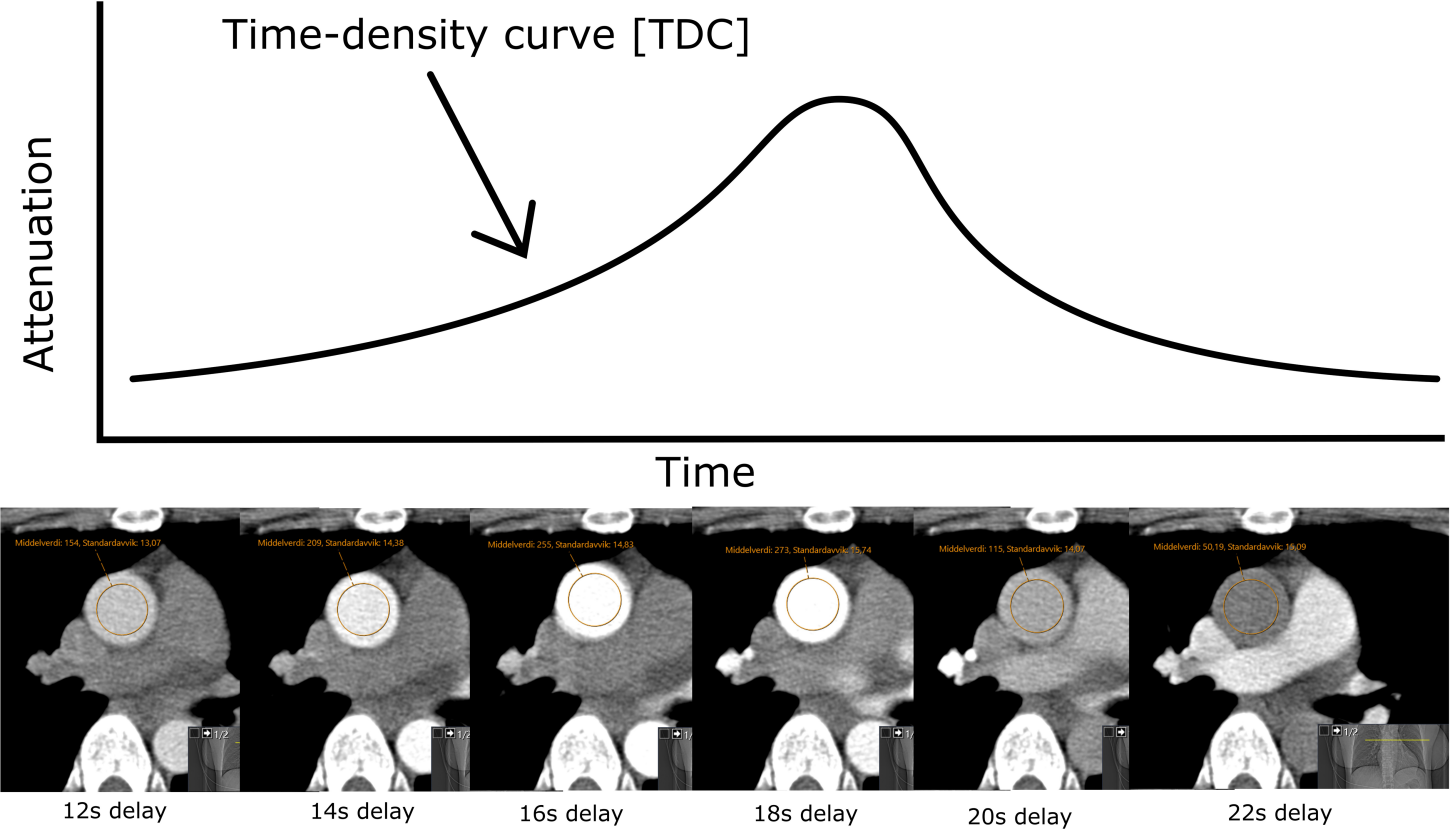
HU measured over time with a ROI with a width of approximately two-third of the aortic diameter, centrally placed in the aorta ascendens, gives a time–density curve.

Test-bolus images were acquired sequentially at 2-s intervals over the aorta, and attenuation in the aorta was measured by a centrally placed region of interest (ROI) with a width of approximately two-third of the aortic diameter (illustrated in Figure 1) on every image acquired in the test-bolus for each patient. The size and placement of the ROI were chosen to minimize the risk of introducing inaccuracies in the HU measurement due to partial volume or movement artifacts.

CCTA was performed as single heartbeat acquisition, prospective acquisition, or retrospective acquisition according to clinical routines. See Table S1 in the Supplementary Material for details. Single heartbeat acquisition was the preferred method, providing a sufficiently regular pulse of approximately 60 bpm or lower. If the participant had an irregular pulse of approximately 60 bpm or lower, prospective CCTA was performed. If single heartbeat acquisition and prospective CCTA were unfeasible, retrospective CCTA was performed.

#### 2.3.2. MRI

Each participant underwent a cardiac MRI immediately after CCTA. Cardiac MRI images were acquired with the Philips Intera 1.5 T (Philips Healthcare, Best, Netherlands). During the cardiac MRI, the participant underwent dynamic functional sequences for volumetric measurement of left ventricular volume throughout the heartbeat. See Figure S1 and Table S1 in the Supplementary Material for details. From these functional sequences, two experienced cardiac radiologists independently calculated the CO using the Philips Intellispace Portal software (Version 10.2, Philips Healthcare, Best, Netherlands), with manually placed endocardial and epicardial contours. The volumetrically measured CO (CO_MR_) calculated by each experienced radiologist was averaged for each participant and used as a method of validation.

### 2.4. Mathematical method for CO estimation

#### 2.4.1. Stewart–Hamilton method

The Stewart–Hamilton method (12, 13) is a method used for estimating CO based on a known amount of tracer injected into the bloodstream. The equation assumes that there is no recirculation of the tracer and that the tracer concentration drops to zero. This never happens due to recirculation, and one needs to extrapolate the curve to zero. Here, we followed the method outlined by Mahnken et al. (14) and fit a gamma variate function to the data using the method described by Madsen et al. (15).

#### 2.4.2. Mathematical compartment model adjusting for patient-specific factors

The total blood volume was calculated with Nadler's formula (16) using height and weight. The mathematical model was based on a compartment model for the distribution of blood in the body, which was suggested by Bae et al. (11). The model was used previously to estimate contrast enhancement in different organs on a group level, and the CO in the original work was predetermined using a correlation (11) based on height and weight. We intentionally disregarded the extracellular space, as its involvement in the distribution of CM was negligible due to the short scan delay in our protocol. Iodinated CM had no relevant interaction with the intracellular space, and it was also disregarded as such (17).

We estimated the CO from the data by fitting the TTP in the model to the TTP in the data. Supplementary Figures S1 and S2 present a typical result. In Supplementary Figure S1, the original model was used, and in Supplementary Figure S2, we changed the injection point to be a plug flow reactor, i.e., similar to a piston-type displacement that more closely resembles the physical situation where a syringe is used to inject the contrast. In addition, we scaled the heart volume proportional to CO divided by the pulse. After these changes to the model, it turned out that the shape of the curve matched the data more closely but the estimated CO was similar as shown in Supplementary Figures S1 and S2. In addition, we adjusted the blood volume and CO for each patient to match the least square estimate of not only the TTP but also all the data. However, this did not lead to any improvement in our estimated CO.

CO_Model_ was compared with CO_MR_, and estimated attenuation in the aorta in HU_Est_ was compared with measured attenuation in the aorta in HU during test-bolus in CCTA (HU_CT_).

### 2.5. Statistical methods

The variables were analyzed using SPSS (IBM SPSS version 26.0.0.1, Armonk, NY, USA). A *p*-value of 0.05 was set as the level of statistical significance. The distributions of the variables were checked for normalcy with Kolmogorov–Smirnov tests. A majority of the variables had a non-normal distribution. Non-normally distributed variables are presented as median, whereas normally distributed variables are presented as average. A correlation was measured with Pearson correlation coefficient *r* and classified as none (*r* = 0.0–0.3), weak (*r* = 0.3 to 0.5), moderate (*r* = 0.5–0.7), strong (*r* = 0.7–0.9), and very strong (*r* = 0.9–1.0) (18).

Using the method of assessing the accuracy of the estimation, and to which degree each variable affected the accuracy, we calculated the relative difference in the estimated CO compared to the CO measured volumetrically with MRI (CO_R−Diff_) (see Equation 1)

(1) $$CO_{R-\mathrm{Diff}}=(CO_{\mathrm{Model}}-CO_{\mathrm{MR}})/CO_{\mathrm{MR}}$$

The participants were stratified into three percentile groups of age (*SPSS -> Transform -> Rank Cases -> Types -> Ntiles*: 3). A selection of variables was assessed with linear correlation (*Analyze -> Regression -> Linear*) with CO_R−Diff_ as the dependent variable. One by one, the variable with the highest variance inflation factor (VIF) above five and lowest statistical significance was removed (19). One by one, the variable with the lowest statistical significance was subsequently removed from the variables with a VIF below five until only statistically significant variables with a low VIF remained.

## 3. Results

Of the 40 included participants, one participant was excluded due to the unacceptable quality of the cardiac MRI, and two outliers were excluded due to an excessive discrepancy in simulated TDC in comparison to measured TDC. See Table 1 for the demographics of the included participants.

**Table 1:** Baseline demographics of participants presented as a statistical overview of the various variables and measurements.

Variable	Baseline demographics of participants										
	Distribution	Mean	Median	SD	95% CI		Gender difference	Signif.	SE	95% CI	
Age (years)	Non-normal	53.16	51.50	13.54	48.65	57.68	Not signif.				
Systolic blood pressure (mmHg)	Non-normal	138.03	137.50	19.00	131.69	144.36	Not signif.				
Diastolic blood pressure (mmHg)	Non-normal	81.73	79.50	10.93	78.09	85.37	Not signif.				
Pulse (bpm)	Normal	59.24	56.50	8.98	56.25	62.24	Not signif.				
Height (cm)	Non-normal	172.89	172.00	10.28	169.46	176.32	15.58	*p* < 0.01	2.21	11.09	20.07
Weight (kg)	Non-normal	84.16	83.50	19.83	77.55	90.77	19.24	*p* < 0.01	5.78	7.50	30.97
Body surface area (m^2^)	Non-normal	1.97	1.98	0.27	1.88	2.06	0.33	*p* < 0.01	0.07	0.19	0.47
Body mass index (kg/m^2^)	Non-normal	27.93	27.60	5.15	26.22	29.65	Not signif.				
Time to peak (s)	Normal	18.32	18.00	2.60	17.46	19.19	Not signif.				
Attenuation in test-bolus (HU)	Non-normal	193.51	176.50	62.35	172.73	214.30	−51.94	*p* < 0.01	18.92	−90.35	−13.52
Simulated attenuation (HU)	Normal	243.65	214.50	85.23	215.23	272.06	−111.76	*p* < 0.01	22.57	−158.64	−64.88
Change in attenuation (HU)	Non-normal	196.11	177.50	59.53	176.26	215.96	−46.92	*p* = 0.02	18.27	−84.01	−9.83
Attenuation in the aorta (HU)	Non-normal	578.57	532.50	194.57	513.70	643.44	−146.73	*p* = 0.02	60.18	−268.91	−24.55
Contrast (ml)	Normal	78.51	80.00	13.53	74.00	83.03	Not signif.				
Saline (ml)	Normal	83.46	80.00	12.00	79.46	87.46	Not signif.				
Contrast dose per body weight (ml/kg)	Non-normal	0.98	0.95	0.24	0.89	1.06	−0.25	*p* < 0.01	0.07	−0.39	−0.11
Measured cardiac output (CO_MR_) (L/min)	Normal	5.05	4.80	1.13	4.67	5.42	0.92	*p* = 0.01	0.34	0.23	1.62
Estimated cardiac output with the compartment model (CO_Est_) (L/min)	Non-normal	3.24	3.30	1.25	1.54	5.51	1.80	*p* < 0.01	0.29	1.22	2.38
Estimated cardiac output with the Stewart–Hamilton method (CO_Est_] (L/min)	Non-normal	9.16	9.06	2.82	4.25	15.36	2.76	*p* < 0.01	0.80	1.14	4.38
Ejection fraction (percent)	Non-normal	63.76	65.00	7.41	51.60	75.00	Not signif.				

Signif., significant.

Men were on average 15.58 cm higher and 19.24 kg heavier than women. They had a 0.33 m^2^ higher body surface area (BSA) and 12.52 higher estimated glomerular filtration rate (eGFR) than those women had. During the CCTA, men received on average 0.25 mg CM less per kilogram of body weight compared to women.

Average pulse during cardiac MRI and CCTA were similar [cardiac MRI average pulse 57.4 (SD 8.49), CCTA average pulse 59.24 (SD 8.98)].

The average CO_MR_ was 5.05 L/min ± 1.13 SD, 95% CI (4.67–5.42). The average CO_Model_ was 3.24 L/min ± 1.25 SD, 95% CI (1.54–5.51). The average CO_SH_ was 9.16 L/min ± 2.82 SD, 95% CI (4.25–15.36). There was a statistically significant difference in average CO_MR_, CO_Model_, and CO_SH_ between men and women, but the difference was more pronounced in CO_Model_ and CO_SH_.

CO_MR_ was 0.92 L/min higher in men than that in women (*p* = 0.01). CO_Model_ was 1.80 L/min higher in men than that in women (*p* < 0.01). CO_SH_ was 2.76 L/min higher in men than that in women (*p* < 0.01).

When stratifying CO_MR_, CO_Model_, and CO_SH_ into quartiles based on BSA, however, there was no statistically significant correlation with gender.

### 3.1. Periprocedural medication

In total, 35 participants received premedication with beta-blockers due to a resting heart rate above 60 bpm. The remaining five participants did not require premedication with beta-blockers or had contraindications. Sublingual nitroglycerine was administered to 39 participants immediately before the main CT scan and cardiac MRI. One participant did not receive sublingual nitroglycerine due to contraindications (systolic blood pressure below 100 mmHg).

### 3.2. CO estimation

#### 3.2.1. CO estimation with the mathematical compartment model

**Figure 2:**
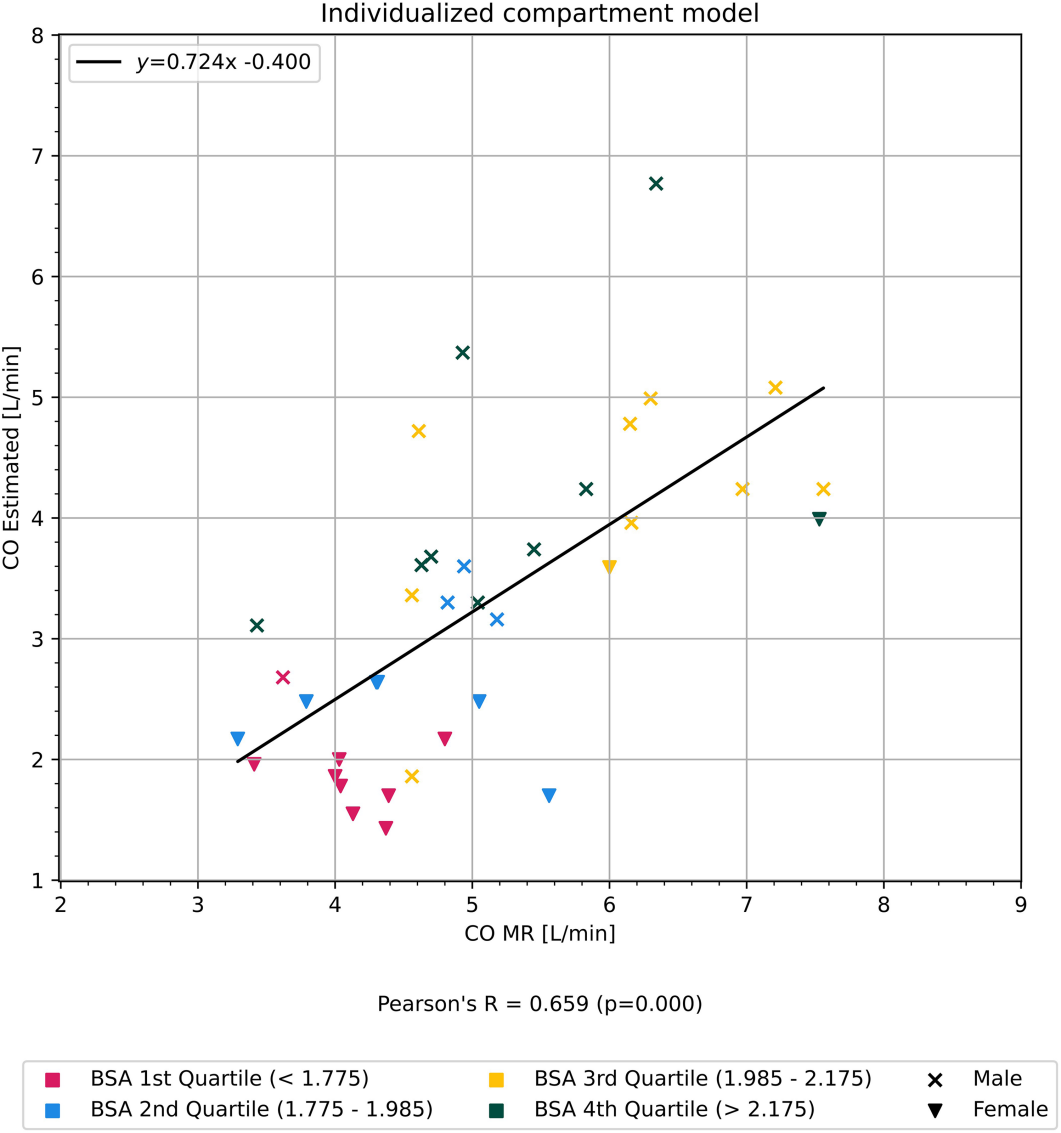
Graph showing correlation of cardiac output estimated using our mathematical compartment model in comparison to cardiac output measured from cardiac magnetic resonance imaging. Participants are colored according to quartiles of body surface area BSA.

CO estimated using the mathematical compartment model (CO_Model_) was moderately correlated with CO_MR_ for all included participants (*r* = 0.66, *p* < 0.01) (Figure 2).

#### 3.2.2. CO estimation with the Stewart–Hamilton method

**Figure 3:**
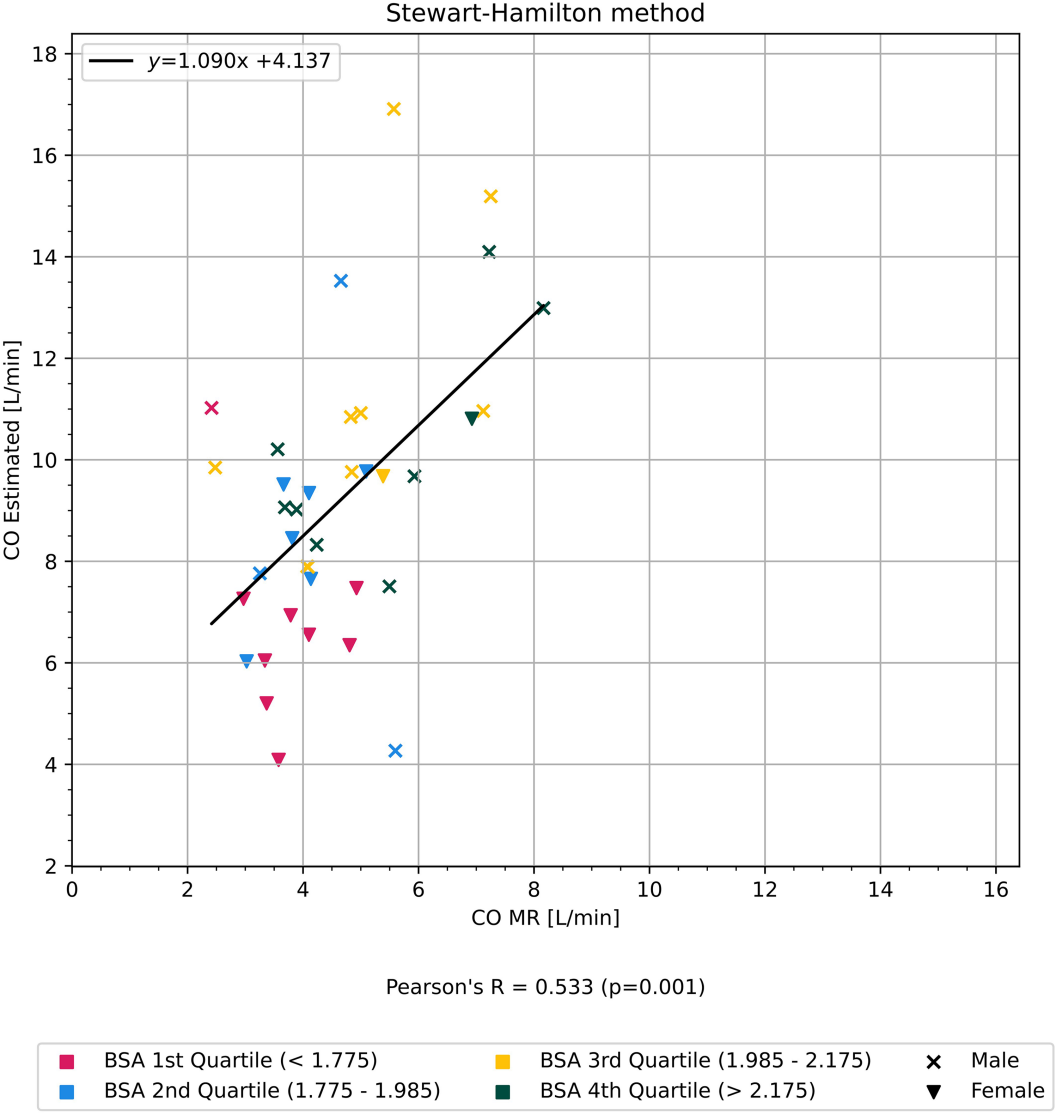
Graph showing correlation of cardiac output estimated using the Stewart-Hamilton method in comparison to cardiac output measured from cardiac magnetic resonance imaging. Participants are colored according to quartiles of body surface area BSA.

CO estimated using the Stewart–Hamilton method (CO_SH_) was moderately correlated with CO_MR_ for all included participants (*r* = 0.53, *p* < 0.01) (Figure 3).

**Figure 4:**
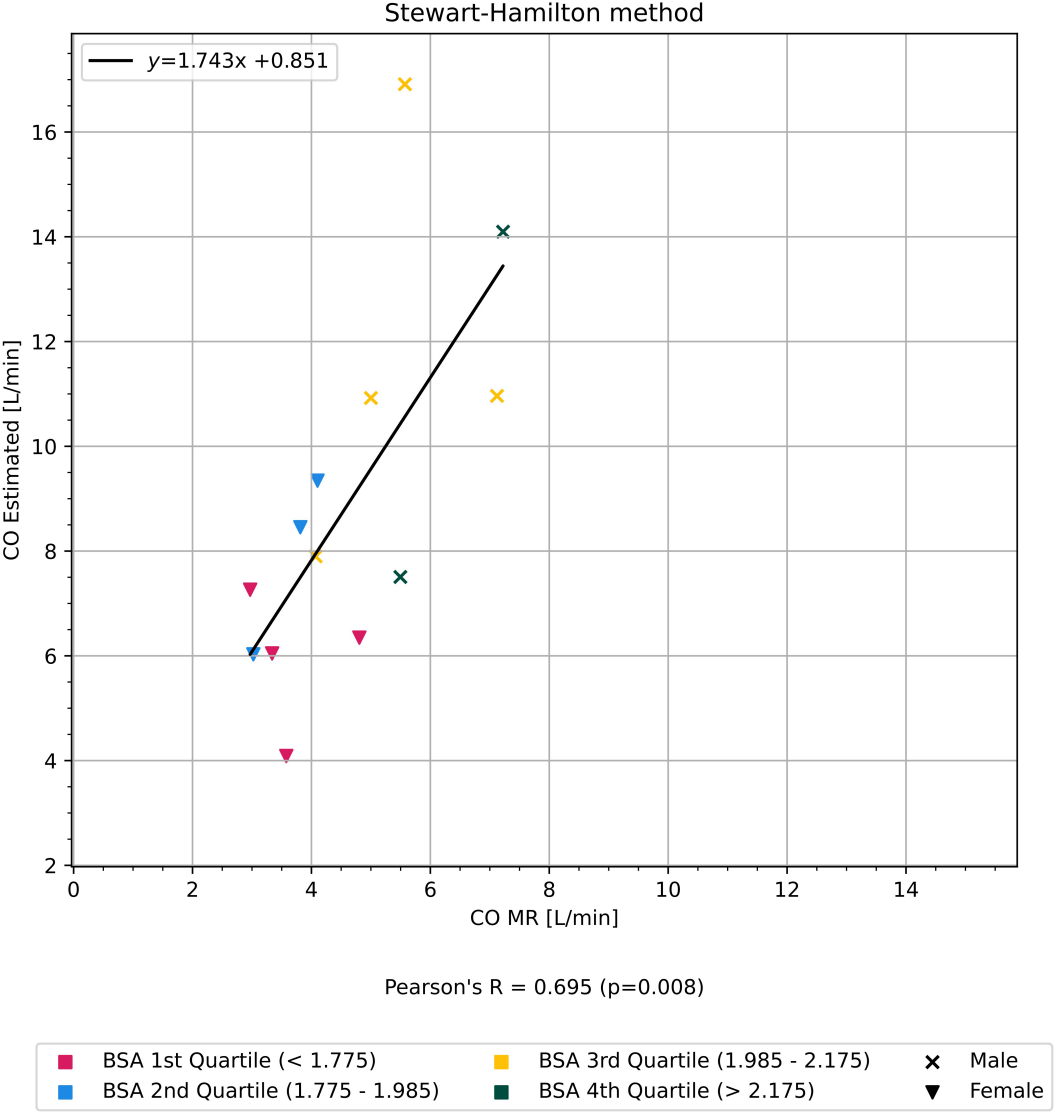
Graph showing the correlation of CO estimated using the Stewart–Hamilton method, but only on a cherry-picked subset of patients with a more complete time–density curve, in comparison to CO measured from cardiac MRI. The participants are colored according to quartiles of BSA.

A subset of participants had a TDC where the HU in the aorta at first image acquisition after scan delay was similar to HU without CM, in addition to scan duration being of sufficient length for HU to normalize. We estimated CO_SH_ in this subset and found a strong correlation with CO_MR_ (*r* = 0.70, *p* < 0.01) (Figure 4).

The patient-specific and scanner-specific variables in this subset of participants did not differ significantly on average from the unselected participants. The only key difference was a completely known vs. parts-unknown TDC.

### 3.3. Statistical analyses

#### 3.3.1. Correlation analyses of specific variables with the accuracy of CO estimation

**Figure 5:**
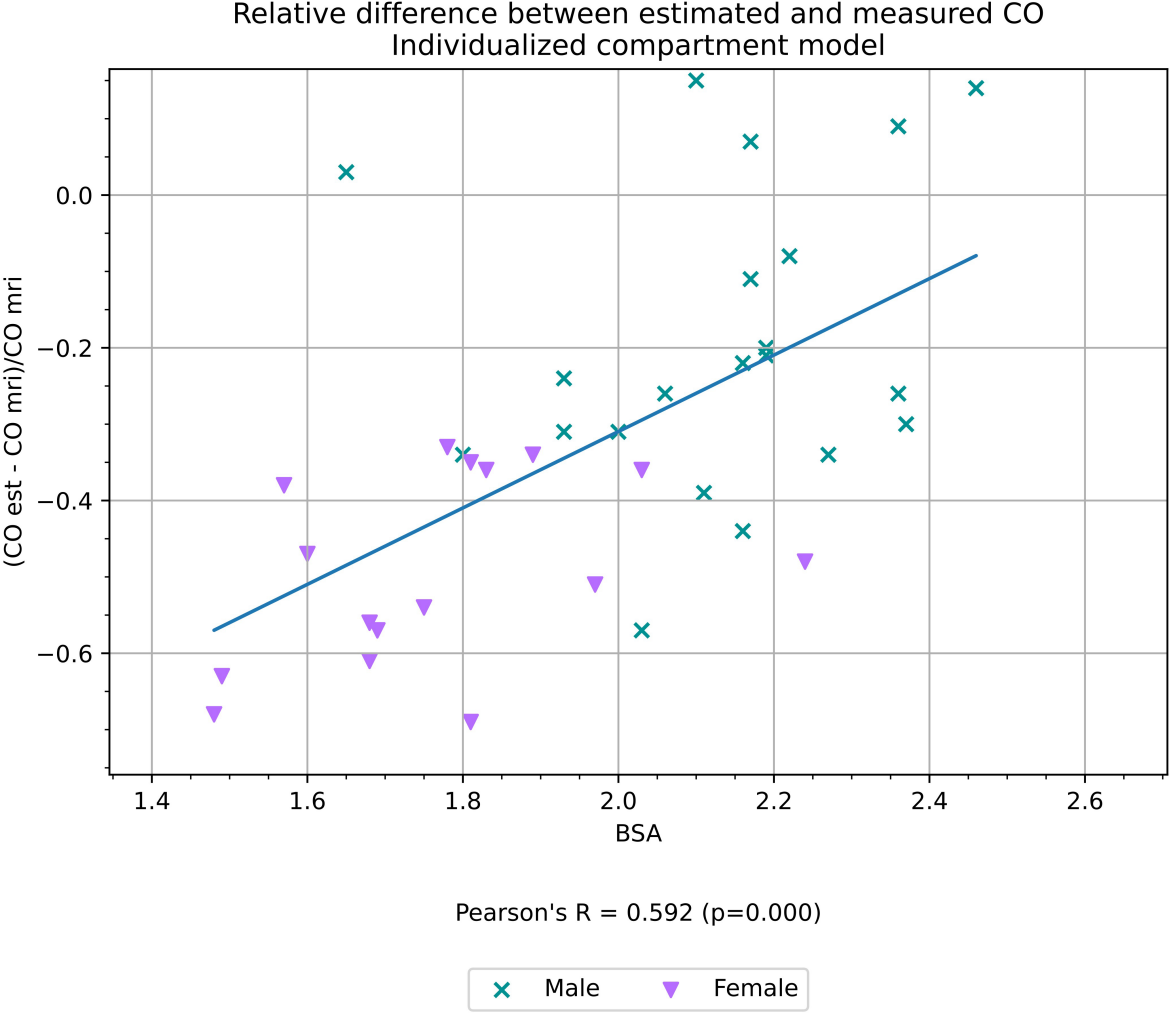
Relative difference between CO estimated with an individualized compartment model and CO measured volumetrically from cardiac MRI, a proxy for the accuracy of the estimation, and its correlation with BSA.

**Figure 6:**
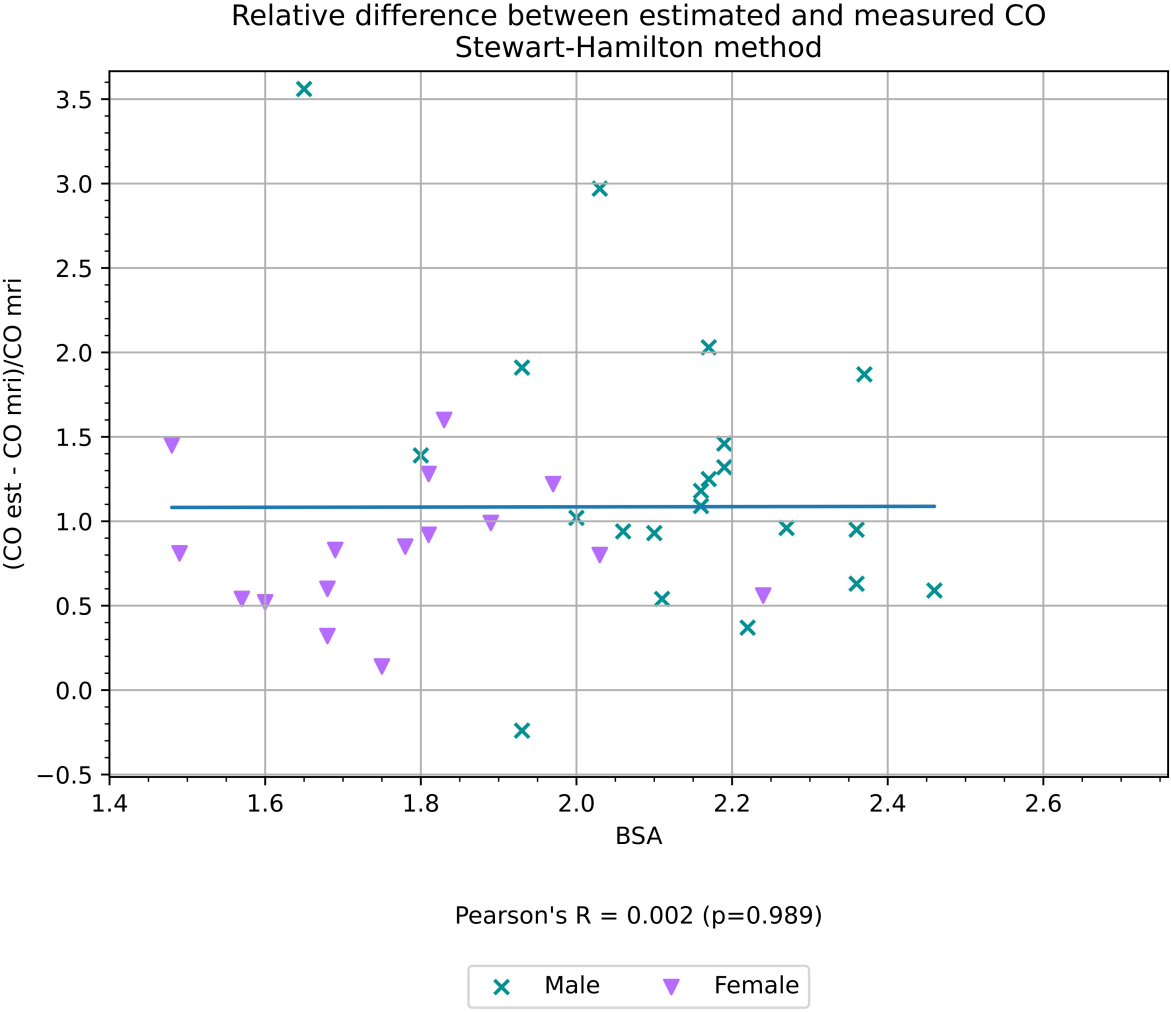
Relative difference between CO estimated using the Stewart–Hamilton method and CO measured volumetrically from cardiac MRI, a proxy for the accuracy of the estimation, and its correlation with BSA.

BSA correlated with the relative difference (see Formula 1) between CO estimated with the compartment model and CO_MR_ (*r* = 0.59, *p* < 0.01) as shown in Figure 5, but did not show any correlation with the relative difference between CO measured with the Stewart–Hamilton model and CO_MR_ (*r* = 0.01, *p* = 0.99) as shown in Figure 6.

**Figure 7:**
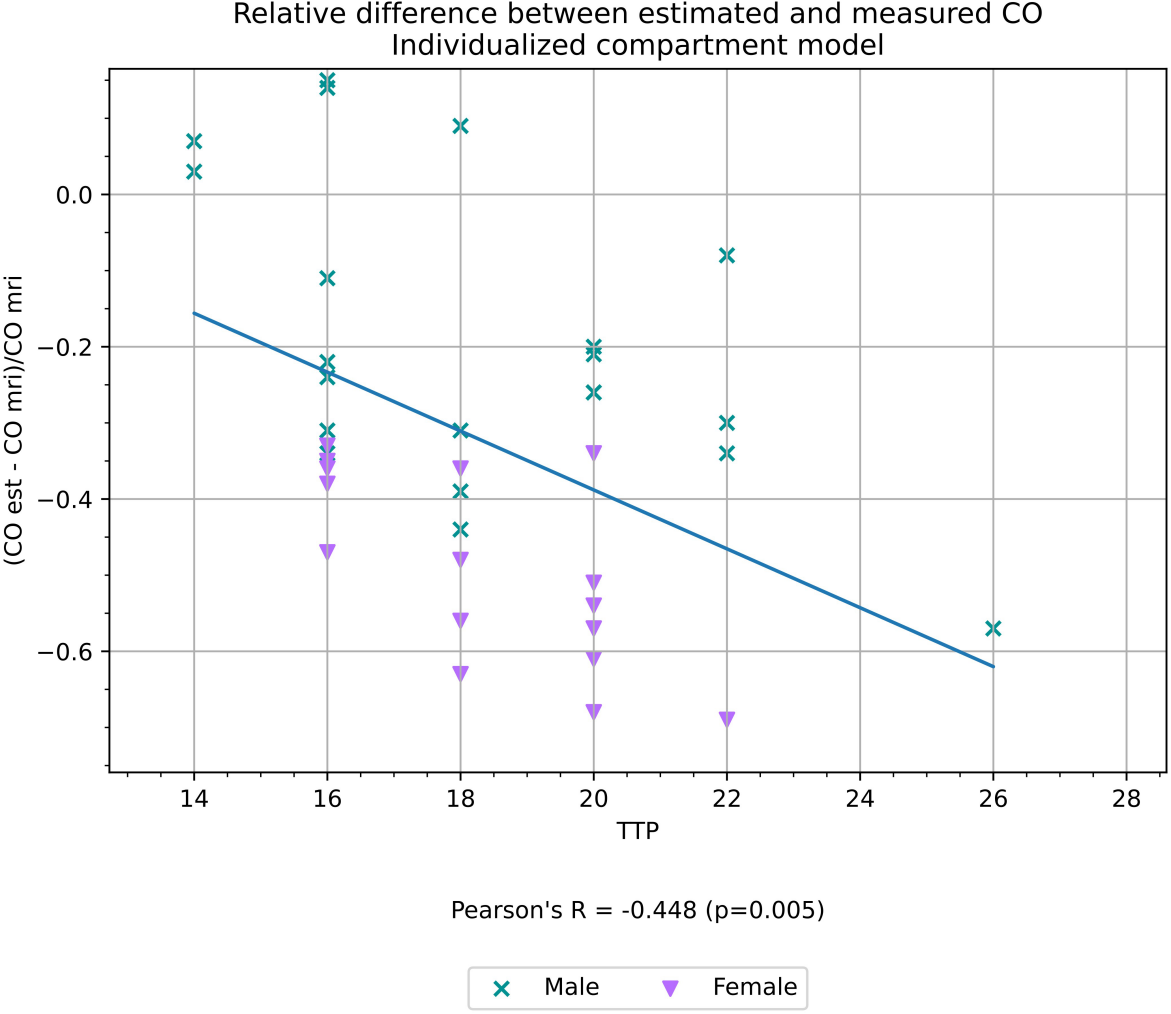
Relative difference between CO estimated with an individualized compartment model and CO measured volumetrically from cardiac MRI, a proxy for the accuracy of the estimation, and its correlation with TTP.

**Figure 8:**
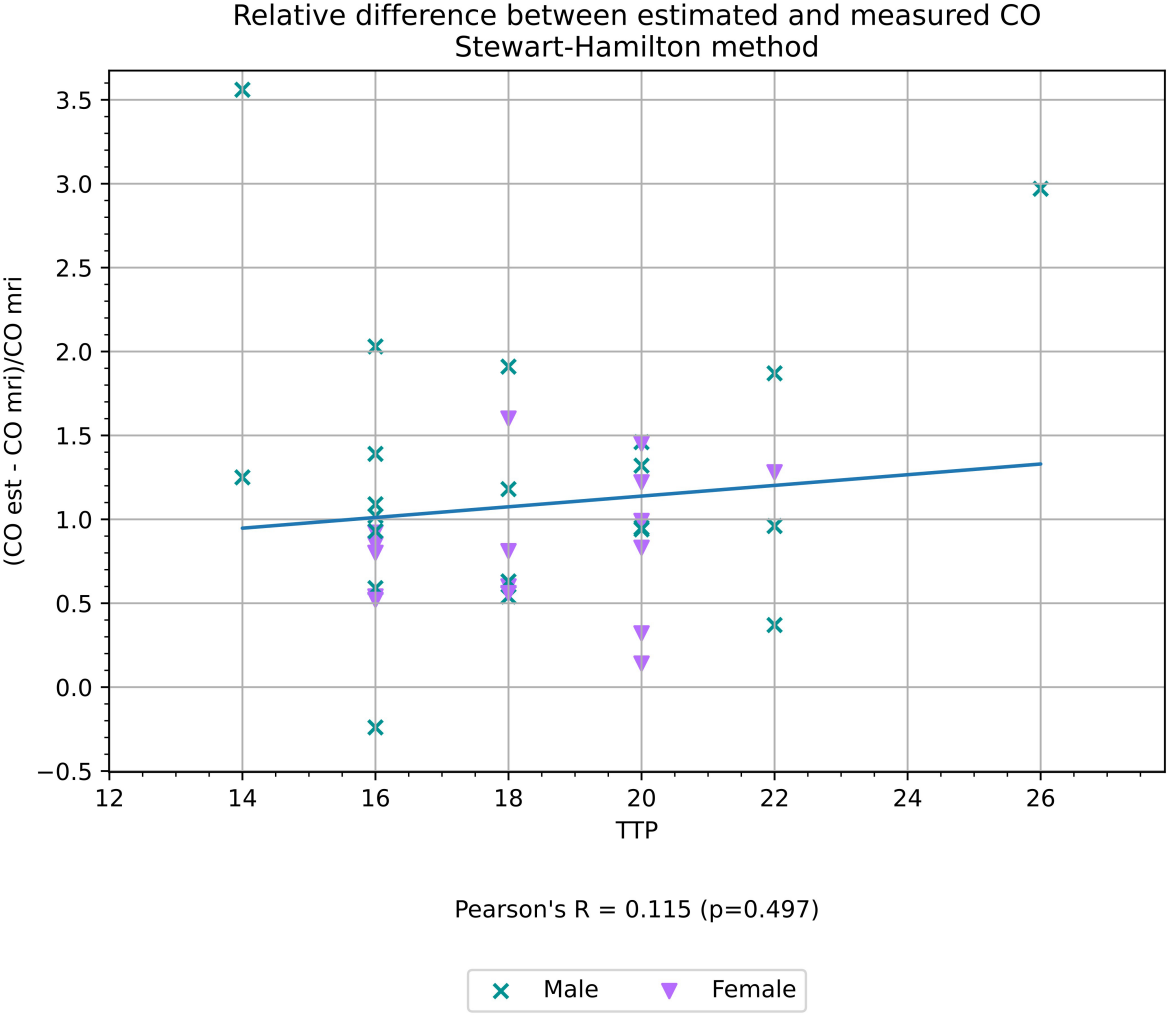
Relative difference between CO estimated using the Stewart–Hamilton method and CO measured volumetrically from cardiac MRI, a proxy for the accuracy of the estimation, and its correlation with TTP.

TTP had a weak negative correlation with the relative difference between CO estimated with the compartment model (*r* = −0.45, *p* < 0.01) as shown in Figure 7, but did not have a statistically significant correlation with the relative difference between CO measured with the Stewart–Hamilton model CO_SH_ and CO_MR_ (*r* = 0.12, *p* = 0.50) as shown in Figure 8.

Age, systolic and diastolic blood pressure, pulse, eGFR, hemoglobin, and CM dose did not have any statistically significant correlation with the relative difference between either method of estimating CO and CO_MR_.

#### 3.3.2. Variance inflation factor

When variance inflation factor (VIF) was analyzed, only TTP (*p* = 0.04, VIF 1.022) and BSA (*p* < 0.01, VIF 1.022) remained statistically significant with a VIF below five.

Gender and the remaining variables, with a statistically significant difference between genders, played no significant role in the accuracy of our model. Their statistical significance, if any, was likely due to covariance according to our analysis.

## 4. Discussion

### 4.1. Summary of main findings

Standardized methods used for estimating CO, such as the Stewart–Hamilton method, are well established (13). In our dataset, the early phase of the TDC, establishing a baseline, is unknown in several patients, and some have insufficient scan duration to obtain the end of the TDC. We propose that this results in the reduced accuracy of the estimated CO using the Stewart–Hamilton method, reducing its generalizability.

Estimated CO from our mathematical compartment model moderately correlated with CO measured from cardiac MRI (Figure 1). In addition, there was a discrepancy in average CO between genders. This was likely due to a compounding effect of statistically significant differences in the height and weight of the participants, and, consequently, BSA, which is directly correlated with the estimation of total blood volume.

In our opinion, the primary benefit of this study pertains to the application of either method to simulate CM distribution in the body and the potential therein to individualize CM dose, rather than replacing cardiac MRI for functional assessment.

### 4.2. Mathematical simulation of contrast distribution in comparison with previous studies

This is, to our knowledge, the first report on estimated test-bolus employing a mathematical compartment model validated against findings of CO from MRI. Our findings are in accordance with findings of a moderately high correlation between estimated CO by CT validated with echocardiography (20).

This work is based on the computer model presented by Bae et al. (10), where a pharmacokinetic model was developed to predict the CT contrast enhancement in different organs. The parameters in the model were based on physiological data from a reference man presented by Leggett et al. (21) The application of the model gives insight into how contrast distribution in the body is affected by injection protocols, height, weight, and sex (22). Sahbaee et al. applied a similar model to assess patient-specific organ dose during a CT examination (23, 24).

When applying the model, one needs to know the CO and the blood volume distribution. The blood volume is usually determined by a correlation between blood volume, height, and weight. We also followed this approach and used Nadler's formula (16).

Previous work has also used a correlation to determine CO with success, as by Mahnken et al. (14), where they found an even higher correlation between test-bolus estimation of stroke volume (SV) determined from test-bolus analysis and geometric analysis with Pearson correlation coefficients of 0.87 and 0.88, respectively. However, this assumption greatly limits the application of these types of models to determine individual injection protocols for CT contrast enhancement.

### 4.3. CO estimation using a compartment model in comparison to the Stewart–Hamilton method

#### 4.3.1. Limitations of the Stewart–Hamilton method

If the complete TDC is known and corrected for contrast enhancement due to recirculation, it can be used to estimate the CO, e.g., by a modified Stewart–Hamilton equation (14). This method, however, necessitates a majority of the TDC to be known to accurately calculate the area under the curve. Our data indicates the importance of measurements in the early phase of the TDC to ensure reliable CO estimation using the Stewart–Hamilton method. Many test-bolus acquisition methods begin after a delay to minimize radiation dose. Subsequently, the beginning curve of the TDC may be unknown.

With our subset of patients and the method with which test-bolus images were acquired, the early phase of TDC is unknown due to scan delay before initial image acquisition to minimize radiation dose. Hence, the weak correlation found between CO estimated with the Stewart–Hamilton method with CO_MR_ in comparison to the results achieved by, for example, Mahnken et al. (14). When selectively applying the method to a subset of participants with a more complete TDC, the correlation improved. There was no significant difference in the demographics between the subset of participants and the unselected participants, further underscoring the importance of a complete TDC as a factor for success when applying traditional methods such as the Stewart–Hamilton method.

Thus, reliably implementing such a method in clinical practice could require an increased dose of radiation by beginning image acquisition immediately after contrast administration, rather than after a delay.

#### 4.3.2. Individualized mathematical compartment model

Our individualized mathematical compartment model provides a CO estimation with easily obtained patient and scanning parameters, which could simplify implementing the method in clinical practice. It may also be more reliable than the Stewart–Hamilton model in instances where the data points in the TDC are limited, especially in regards to the early phase of the TDC, as is the case with our data.

### 4.4. Gender discrepancy

The statistical difference in CO_MR_ and CO_Model_ between genders is expected, based on the demographics of the population from which our patients were selected (25, 26). Gender discrepancy is due to differences in height and weight, confirmed by the lack of statistically significant correlation with gender and CO_MR_ or CO_Model_, when stratified into quartiles based on BSA.

Although there was a statistically significant difference in eGFR between the genders, the role of renal filtration and its impact on the CM removal from the blood is negligible when scanning during the arterial phase. This was further underlined by the lack of statistical significance and a high VIF of eGFR, both in relation to CO_MR_ directly, and the relative difference between estimated CO and CO_MR_ (see Formula 1).

### 4.5. The role of BSA in the distribution of estimated blood volume

Our computational model uses height and body weight to estimate total blood volume according to Nadler's formula (16). The difference between average CO_Model_ and CO_MR_ could be caused by the model overemphasizing the estimated total blood volume.

While the estimation of total blood volume could indeed be correct, there may be less variance in central blood volume, specifically in the aorta and pulmonary circulation based on BSA, and a larger variance in peripheral blood volume.

### 4.6. Multivariate correlation with the precision of the mathematical compartment model and compounding factors

A compounding factor could be that the routine contrast administration protocols utilized at our department are generalized, and as a result, women received on average 0.25 ml/kg higher dose of CM than what men received, which is a statistically significant difference in dosage (*p* = 0.01, 0.07 SE, 0.11–0.39 95% CI). The generalized CM protocol based on body weight is, however, in line with accepted clinical practice in use at other departments of radiology (27).

The correlation of BSA (Figure 5), with the relative difference between CO_Model_ and CO_MR_ (detailed in Formula 1) and the high VIF and low statistical significance of gender in multivariate correlation analysis supports our supposition that gender itself is not a significant factor for simulation.

The blood volume distribution in our mathematical model is built on the reference man model as outlined by Bae (5). Our data indicate the potential overemphasis of BSA in our mathematical compartment model, and there may be a smaller variation in central blood volume based on BSA in comparison to peripheral blood volume.

### 4.7. Strengths and limitations

As a proof of concept, this study aimed to compare the application of a compartment model to the more established Stewart–Hamilton method. One limitation is the small number of included participants. However, the amount of included participants is comparable to that in similar studies performed by, for example, Bae et al. (10) and Mahnken et al. (14).

The individualized mathematical model was validated against cardiac MRI, the current non-invasive gold standard for CO assessment (28).

The participants were included prospectively and consecutively from clinically referred patients who gave informed consent. A possible limitation is that 39 of the 40 included patients were Caucasian and one was of Latino descent, all of which reside in the same region. As such, our model may not be representative of, nor directly applicable to, other ethnic groups or regions.

By performing the cardiac MRI immediately after CCTA, we sought to mirror the dose of long-acting beta-blockers between the two examinations as closely as possible. It was, however, not feasible to ensure that the serum level of beta-blockers was identical, which is a possible limitation.

### 4.8. Conclusion

The TDC acquired during CCTA can be used to estimate CO. The Stewart–Hamilton method and our mathematical compartment model both show moderate correlation when applied to our data. Each method has various strengths and limitations, which may significantly affect its reliability. If the majority of the TDC is known, the Stewart–Hamilton method may provide a reliable CO estimation. However, as is the case with our data, the lack of data points in the TDC reduces the reliability of the Stewart–Hamilton method.

Integrating patient-specific, contrast-specific, and scan-specific parameters in a mathematical compartment model provides an alternative non-invasive method of estimating CO from CCTA, which may prove to be more reliable than the Stewart–Hamilton method in certain applications.

Regardless, both methods show great potential for application in clinical practice, and considering clinical guidelines increasingly recommend CCTA for the assessment of chest pain, reliable CO estimation from CCTA would further increase its diagnostic value.

## Data Availability

The original contributions presented in the study are included in the article/**Supplementary Material**, further inquiries can be directed to the corresponding author.

## Supplementary material

Supplementary Figure 1 Graph showing time to peak fit to the data for patient 6 using the original model described by Bae et al.

Supplementary Figure 2 Graph showing time to peak fit to the data for patient 6 using our modified model.
