# Supplementary material for: Estimating cardiac output from coronary CT angiography: an individualized compartment model in comparison to the Stewart–Hamilton method
Source: Front Cardiovasc Med. 2023 Nov 20;10:1156332. doi: 10.3389/fcvm.2023.1156332 (PMC10694230; doi:10.3389/fcvm.2023.1156332)
Supplement: Supplementary file 1 [file Table1.docx]

Table 1 – Scan parameters and details pertaining to coronary computed tomography angiography techniques.

| ***Scan parameters for coronary computed tomography angiography techniques*** | | | | |
| --- | --- | --- | --- | --- |
| *Scan Parameters* | *Test-bolus* | *Single heartbeat acquisition* | *Prospective* | *Retrospective* |
| *Tube voltage* | 100 kV | 100 kV | < 90 kg: 100 kV  > 90 kg: 120 kV | < 90 kg: 100 kV  > 90 kg: 120 kV |
| *Milliamperage* | 45 mAs | 270 mAs | 230 mAs | 200 mAs |
| *Rotation time* | 0.28 s | 0.28 s | 0.28 s | 0.28 s |
| *Slice Collimation* | Single 10 mm slice | 3 mm 0.6 mm collimation | 3 mm 0.6 mm collimation | 3 mm 0.6 mm collimation |
| *Pitch* | None | 34.5 | 34.5 | 0.17 |
| *Injection rate* | 6 ml/s | 6 ml/s | 6 ml/s | 6 ml/s |
| *Contrast* | 20 ml contrast  40 ml saline | 80 ml contrast  80-100 ml saline | See comment* | See comment** |
| *Start Delay* | 12 s | Time to peak of test-bolus plus 5 seconds | Time to peak of test-bolus plus 2 seconds | Time to peak of test-bolus plus 2 seconds |
| ** - Contrast dose determined by total scan time by calculating the sum of contrast medium and saline to ensure coverage of the entire scan time. For example: 80 ml contrast at 6 ml/s equals 13.3 seconds, 100 ml saline at 6 ml/s equals 16.7 seconds, totaling 30 seconds scan time. If scan time differs, adjust the amounts accordingly.* | | | | |
| *** - Contrast dose is determined by multiplying flow rate (6 ml/s) by scan time and adding an additional 10 ml of contrast. For example: (8 seconds scan time * 6 ml/s) + 10 ml = 58 ml. Injection time must be minimum 10 seconds. Saline to “flush” contrast is 4:1 ratio compared to contrast dose.* | | | | |
| *Scan parameters for various CCTA techniques contained within local electronic quality control system used by radiographers for image acquisition.* | | | | |
