# Supplementary material for: Estimating cardiac output from coronary CT angiography: an individualized compartment model in comparison to the Stewart–Hamilton method
Source: Front Cardiovasc Med. 2023 Nov 20;10:1156332. doi: 10.3389/fcvm.2023.1156332 (PMC10694230; doi:10.3389/fcvm.2023.1156332)
Supplement: Supplementary file 2 [file Table2.docx]

Table 2 – Parameters for cardiac magnetic resonance imaging sequences used for volumetric calculation of left ventricular function including cardiac output.

| ***Parameters for volumetric functional cardiac magnetic resonance imaging sequences*** | |
| --- | --- |
| *Anatomical sequences* | Balanced long-axis breath-hold turbo field echo cine |
|  | Balanced 3-chamber breath-hold turbo field echo cine |
|  | Balanced short-axis breath-hold turbo field echo cine |
|  | Balanced 4-chamber breath-hold turbo field echo cine |
| *Functional sequences* | Balanced short-axis breath-hold turbo field echo cine  (18-20 slices, 8 mm slice thickness, 0 gap, 30 phases per slice) |
| *Volumetric cardiac MRI sequences. Anatomical sequences obtained for accurate orientation of functional sequence.* | |
