# Supplementary figures and images for: Estimating cardiac output from coronary CT angiography: an individualized compartment model in comparison to the Stewart–Hamilton method
Source: Front Cardiovasc Med. 2023 Nov 20;10:1156332. doi: 10.3389/fcvm.2023.1156332 (PMC10694230; doi:10.3389/fcvm.2023.1156332)

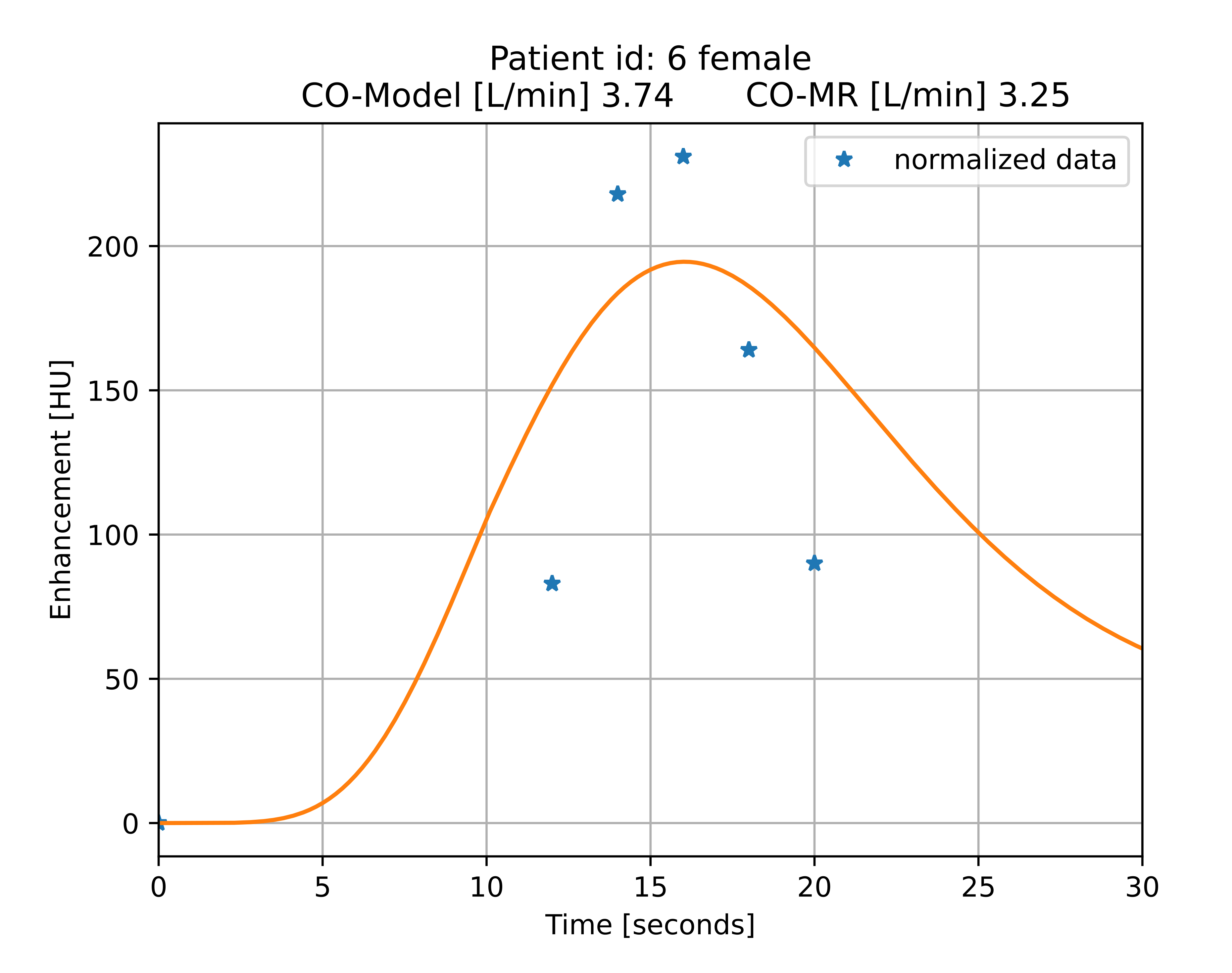

Supplement: Supplementary Figure 1 — Graph showing time to peak fit to the data for patient 6 using the original model described by Bae et al. [file Image1.png]

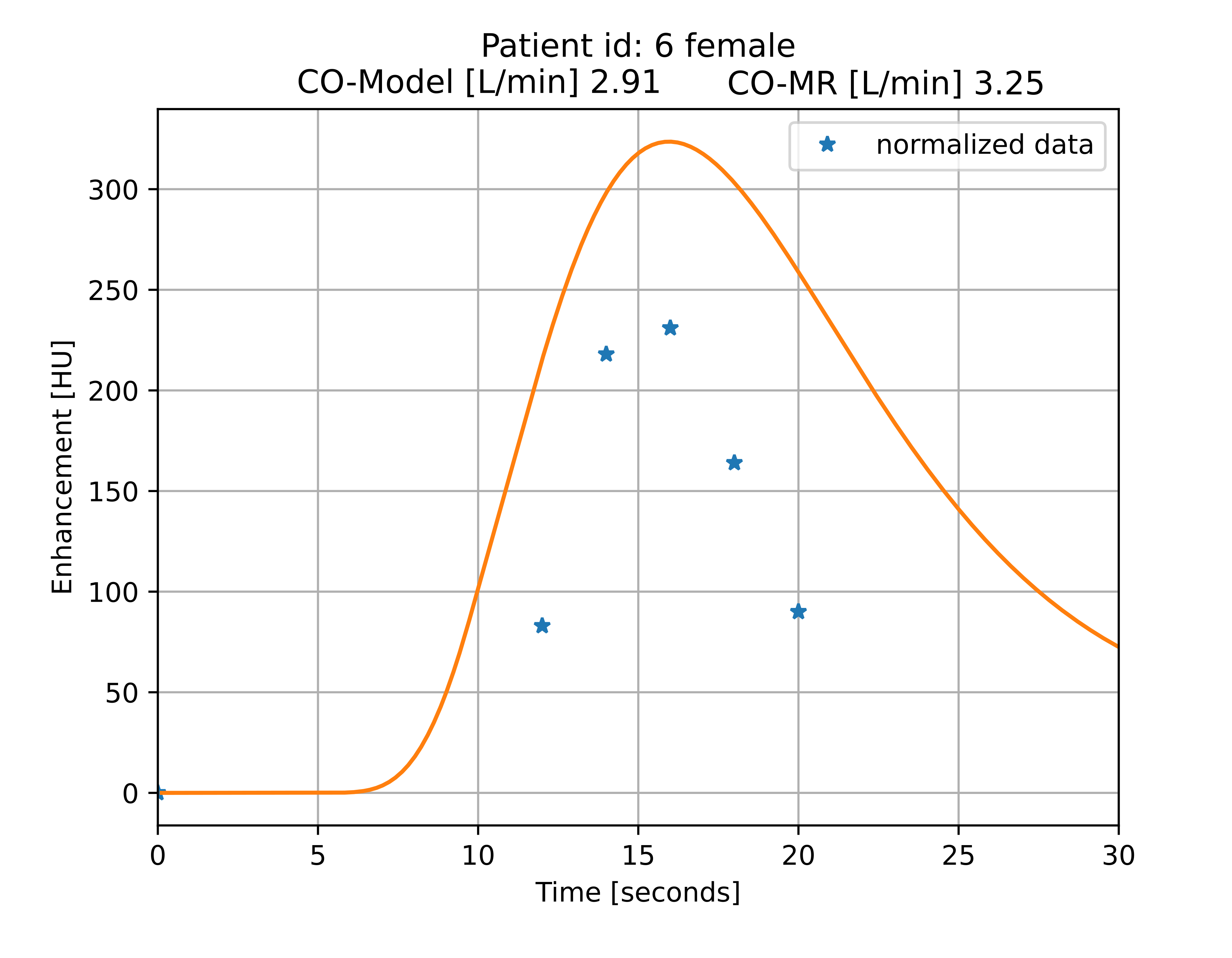

Supplement: Supplementary Figure 2 — Graph showing time to peak fit to the data for patient 6 using our modified model. [file Image2.png]
